# The Role of *Carboxydothermus hydrogenoformans* in the Conversion of Calcium Phosphate from Amorphous to Crystalline State

**DOI:** 10.1371/journal.pone.0089480

**Published:** 2014-02-26

**Authors:** Mathieu Haddad, Hojatollah Vali, Jeanne Paquette, Serge R. Guiot

**Affiliations:** 1 Energy, Mining and Environment Portfolio, National Research Council Canada, Montreal, Quebec, Canada; 2 Department of Microbiology, Infectiology and Immunology, Université de Montréal, Montreal, Quebec, Canada; 3 Department of Earth and Planetary Sciences, McGill University, Montreal, Quebec, Canada; 4 Facility for Electron Microscopy Research, McGill University, Montreal, Quebec, Canada; University of South Florida College of Medicine, United States of America

## Abstract

Two previously unknown modes of biomineralization observed in the presence of *Carboxydothermus hydrogenoformans* are presented. Following the addition of NaHCO_3_ and the formation of an amorphous calcium phosphate precipitate in a DSMZ medium inoculated with *C. hydrogenoformans*, two distinct crystalline solids were recovered after 15 and 30 days of incubation. The first of these solids occurred as micrometric clusters of blocky, angular crystals, which were associated with bacterial biofilm. The second solid occurred as 30–50 nm nanorods that were found scattered among the organic products of bacterial lysis. The biphasic mixture of solids was clearly dominated by the first phase. The X-ray diffractometry (XRD) peaks and Fourier transform infrared spectroscopy (FTIR) spectrum of this biphasic material consistently showed features characteristic of Mg-whitlockite. No organic content or protein could be identified by dissolving the solids. In both cases, the mode of biomineralization appears to be biologically induced rather than biologically controlled. Since Mg is known to be a strong inhibitor of the nucleation and growth of CaP, *C. hydrogenoformans* may act by providing sites that chelate Mg or form complexes with it, thus decreasing its activity as nucleation and crystal growth inhibitor. The synthesis of whitlockite and nano-HAP-like material by *C. hydrogenoformans* demonstrates the versatility of this organism also known for its ability to perform the water-gas shift reaction, and may have applications in bacterially mediated synthesis of CaP materials, as an environmentally friendly alternative process.

## Introduction

Biomineralization as described by Lowenstam [1] is the ability of living organisms to form minerals as well as materials composed of an organic and inorganic phase [2], [3]. Among more than 60 biominerals formed by bacteria discovered so far, 25% are amorphous and 75% crystalline. Several authors [3]–[5] have investigated the mechanism of biomineralization and found that organisms across different phyla control biomineralization in a distinct manner and that biominerals have different functions. According to Mann [6] biomineralization occurs at the organic-inorganic interface where a molecular recognition system is involved in the control of crystal nucleation and growth.

Biomineralization processes fall in two categories: biologically induced mineralization (BIM) and biologically controlled mineralization (BCM) [1]. In BIM, biomineralization occurs outside the cell and none of the cell components are serving as a template for nucleation and growth of the precipitate. In this case, cellular activity results in changes in the microenvironment and anionic and cationic precipitation [3]. Biominerals produced by BIM are characterized by poor crystallinity and high variations in morphology, water content, structure, particle size as well as the presence of trace elements [7]. In BCM, also known as inorganic matrix-mediated mineralization [1], the cell controls all of the above described stages of mineralization from nucleation to crystal-formation, leading to a highly specie-specific product [8]. BCM is based on a site-specific matrix (cytoplasm or on the cell wall) that enables the formation of a compartmentalized environment with its own chemical composition. Nucleation is then made possible by sequestering specific ions leading to supersaturation and precipitation in the matrix [9].

Bacteria living under high temperature conditions are known as thermophiles (40–69°C) and hyperthermophiles (70–110°C). Biomineralization processes in this latter group of bacteria have not been extensively explored yet. Indeed, known processes describe magnetite and realgar formation [10] as well as reductive precipitation of uranium, manganese and other toxic metals [11].

In this study, we report that *C. hydrogenoformans* a carboxydotrophic hydrogenogenic hyperthermophilic bacterium [12] converts an amorphous calcium phosphate phase into a fully crystalline whitlockite mineral and spherulitic clusters that we interpret to be hydroxyapatite-like nanocrystals. In addition to conventional microbiological analysis, Fourier transform infrared spectroscopy (FTIR), X-ray diffractometry (XRD) and electron microscopy techniques were applied. We demonstrate that an abiotic soluble CaP precursor is converted, in the presence of an active culture of *C. hydrogenoformans*, to a biphasic mixture of granular aggregates of whitlockite and spherulitic hydroxyapatite. This phase is then converted in the crystalline whitlockite by *C. hydrogenoformans* activity.

Microbial calcification is a widespread phenomenon, which includes the formation of phosphate salts of calcium (CaP) [13]. CaP displays high biocompatibility and biodegradability due to their chemical similarity to calcified tissue [14]–[16]. A large range of CaP, which differ in origin, composition and form, are currently used in medicine for regeneration of hard tissues [17]. Depending on the required characteristic (bioactive or resorbable material) for CaP applications (bone replacement, filling or coating, functionalized nanoparticle), different phases of CaP ceramics are used (β-tri-calcium phosphate (β-TCP), hydroxyapatite or biphasic CaP) [15], [18]. The chemical synthesis of CaP and CaP-based materials, while being very effective on one hand, are relatively expensive and eco-hazardous, requiring extremes of temperature and pH [14]. Thus, the present work offers an alternative biological approach with a more environmentally friendly process making *C. hydrogenoformans* a possible ecofriendly nanofactory for CaP synthesis.

## Materials and Methods

### Bacterial strain and growth conditions

*C. hydrogenoformans* (DSM 6008) was obtained from the German Collection of Microorganisms and Cell Cultures (DSMZ, Braunschweig, Germany). Microorganisms were cultivated under strictly anaerobic conditions in basal mineral bicarbonate-phosphate buffered medium that contained (in g/L of demineralized water): KCl (0.33), MgCl_2_⋅6H_2_O (0.52), CaCl_2_⋅2H_2_O (0.29), NH_4_Cl (0.33), KH_2_PO_4_ (0.33). The medium was supplemented with 10 mL⋅L^−1^ of trace metals solution. The medium was boiled and then introduced anaerobically in sterilized serum bottles under N_2_ air flush. After autoclaving, it was then complemented with (in mL⋅L^−1^ of medium): 5% NaHCO3 stock solution (20), 2.5% Na2S⋅9H2O stock solution (10), 0.5% yeast extract solution (10) and vitamin solution (1). The trace metals and vitamin stock solutions were prepared as described elsewhere [19]. All stock solutions were autoclaved, except the vitamin solution, which was sterilized by filtration through 0.22 µm filter membranes. After complementation, the pH was between 6.8 and 7.0. All experiments were carried out at 70°C, 150 rpm in 500 mL bottles. Bottles contained 200 mL of medium inoculated with the same amount of biomass under a 300 mL headspace. Initial headspace composition was set at 100% CO and 1 atm.

### Control experiments

In control experiments, the bacterial biomass was resuspended in a modified medium described by Zhao and coll. [20] in which no precipitation of amorphous calcium phosphate was observed. The modified medium differed from the DSMZ one only in MgCl_2_⋅6H_2_O, CaCl_2_⋅2H_2_O, KH_2_PO_4_ and NaHCO_3_ concentrations, which were (in g⋅L^−1^ of demineralized water): 0.102, 0.015, 0.136, 0.401, respectively. In that medium, no amorphous CaP was observed to form abiotically over a period of 30 days, and the addition of a live bacterial culture did not induce detectable precipitation of CaP. The modified medium was also used to determine the proteomic profile of *C. hydrogenoformans* when no biomineralization took place (see biomolecular techniques).

Another control experiment was conducted to verify if proteins or amino acids released in the medium by the bacteria had a direct or indirect role in the crystallization of the precipitate. Dry amorphous precipitate obtained in the sterile DSMZ medium was incubated for 15 days at 70°C in the filtered (0.33 µm) inoculated DSMZ medium from which crystalline phases had been recovered. The result of this control experiment was also negative as XRD analysis showed that the CaP precipitate remained amorphous.

In order to exclude the precipitation of an amorphous calcium carbonate in the DSMZ medium, the third abiotic control experiment was carried out using NH_3_OH as buffer instead of NaHCO_3_. A similar precipitate appeared and its energy dispersive X-ray spectrometry (EDX) patterns showed Ca and P peaks identical to those of the solid produced by NaHCO_3_ addition. This confirmed that the amorphous precipitate formed in the DSMZ medium was dominantly a calcium phosphate phase.

### Sampling procedures

All measurements that were carried out on the DSMZ medium were processed immediately after sampling in order to avoid any time related alteration. For precipitate characterization, samples were first concentrated by centrifugation during 10 min at 15000 rpm. Supernatant was removed and the pellet washed 3 times in MilliQ water to remove any remaining of the medium. Since the abiotic precipitate obtained in the absence of *C. hydrogenoformans* was highly soluble in water, the pellet obtained by centrifuging the control samples was washed only once in MilliQ water prior to any characterization.

### Experimental parameters

#### Dissolved total phosphate

Dissolved phosphate ions concentration was measured on aliquots sampled from bottles inoculated with active cultures of *C. hydrogenoformans*. Sterile control series were also conducted on DSMZ medium. 2 mL of medium was sampled every 24 hours and centrifuged. Supernatant was analyzed on a Hamilton PRP-X100 (Hamilton Company, Reno, NV, USA) polymer-based chromatography column (250×41 mm O.D.) in a high-performance liquid chromatograph TSP model P4000 & AS 3000 (TSP, San Jose, CA, USA). Conductivity data were obtained by using a Waters Millipore detector model 432. The mobile phase was p-hydroxybenzoic acid at pH 8.5 with 2.5% methanol at a flow rate of 1.8 mL.min^−1^ at 40°C.

#### Organics and Inorganics

The suspended solids (SS) and volatile suspended solids (VSS) were determined according to Standard Methods [21]. The sample was dried at 105°C over night, weighed then placed in a muffle furnace at 600°C for two hours. VSS is determined from the weight loss from ignition.

#### Volatile fatty acids (VFA)

VFAs (i.e. acetic, propionic and butyric acids) were measured on an Agilent 6890 (Wilmington, DE) gas chromatograph (GC) equipped with a flame ionization detector (FID) on 0.2 µl samples diluted 1∶1 (vol./vol.) with an internal standard of iso-butyric acid in 6% formic acid, directly injected on a glass column of 1 m×2 mm Carbopack C (60–80 mesh) coated with 0.3% Carbowax 20 M and 0.1% H_3_PO_4_. The column was held at 130°C for 4 min. Helium was the carrier gas fed at a rate of 20 mL⋅min^−1^. Both injector and detector were maintained at 200°C.

#### Solvents

For measurement of solvents (methanol, ethanol, acetone, 2-propanol, tert-butanol, n-propanol, sec-butanol, n-butanol) 100 µL of liquid was transferred into a vial that had 20 mL of headspace and was crimp sealed with a Teflon-coated septum. The vial was heated at 80°C for 2 min, then 1000 µl of headspace gas was injected onto a DB-ACL2 capillary column of 30 m×530 mm×2 µm using a Combipal autosampler (CTC Analytics AG, Zwingen, Swizerland). The column was held at 40°C for 10 min. Helium was the carrier gas at a head pressure of 5 psi. The injector and the detector were maintained at 200°C and 250°C, respectively.

#### Mono and disaccharides

Mono and disaccharides were measured using an HPLC from Waters Corporation (Milford, MA) consisting of a pump (model 600, Waters Corporation) and an auto sampler model 717 Plus equipped with a refractive index detector (model 2414, Waters Corporation). Organics acids are monitored using a PDA detector (model 2996, Waters Corporation). The column used for the separation is Transgenomic ICSep IC-ION-300 (300 mm×7.8 mm OD) (Transgenomics, San Jose, CA, USA). The mobile phase is 0.01N H2SO4 at 0.4 mL min^−1^. Analysis is carried out at 35°C.

### Sample characterization

The goal of sample characterization was to compare the solid precipitate obtained from experiments carried out in inoculated and sterile DSMZ media. The characterized solid was obtained by centrifugation of the sampled medium and could not be physically separated from the biomass. In some cases, the bacterial biomass was eliminated by calcination (heating to 600°C for 2 hours) but most observations were carried out on a dried (105°C overnight) composite material made of bacterial biomass intimately mixed with the CaP precipitate. Precipitates from inoculated medium (dried and calcinated to remove all organic matter) and from sterile medium (dried only) were analyzed by XRD. Number and positions of XRD peaks were unchanged from dried-only to dried and calcinated precipitates. Also, XRD patterns of dried precipitate from the sterile medium consistently showed broad humps of an amorphous material, showing that drying did not induce crystallinity. To eliminate the signature of the biomass, FTIR and scanning electron microscopy (SEM) analysis were conducted on the calcinated and dried sample from inoculated media, and compared to those of dried-only samples from sterile media.

#### Elemental analysis of the biotic precipitate

Elemental analysis was performed on a dry sample of a 30-day culture of *C. hydrogenoformans* in the DSMZ medium. Standard Methods were used for determination of elemental carbon, hydrogen, nitrogen, oxygen and sulfur [22] [23]. The sample was combusted at 1030°C. The combustion gases produced are then passed on a GC (ECS 4010, Costech Analytical Technologies, Valencia, CA) using ultra high purity helium as the carrier gas and equipped with a TCD, which analyzes the concentrations of CO_2_, N_2_, H_2_O and SO_2_ from which percentages of carbon, hydrogen, nitrogen and sulfur are derived. The same procedure was utilized for oxygen analysis using a combustion elemental analyzer EA 1108 (Fisons/Carlo Erba, Milan, Italy). Similar samples were analyzed at two different analytical facilities (Dept. of Chemistry, Université de Montréal, Montreal, QC and Chemisar Inc., Guelph, ON) and resulted in the same elemental content.

#### Metals and phosphorus content of the biotic precipitate

A centrifuged sample from a 39 days *C. hydrogenoformans* culture was washed twice and resuspended in milliQ water. Phosphorus was determined using colorimetric methods (method 365.1, [24]). Calcium and metals were determined by Agatlabs Inc. (Montreal, QC) using inductively coupled plasma mass spectrometry (Elan 9000, Perkin-Elmer, Überlingen, Germany) [25].

#### X-ray diffractometry of the abiotic and biotic precipitate

Phase analysis was performed on a Bruker D8 Advance X-ray diffractometer (Bruker, Germany) using Cu Kα radiation (1.5417Å) at 40 kV and 40 mA. The scanning range (2θ) was from 5° to 80° at a scan speed of 0.15° min^−1^ (for the dried sample) and 0.075° min^−1^ (for the calcinated sample) with a step size of 0.025°.

Phases were identified by matching the peaks to the JCPDS (Joint Committee on Powder Diffraction Standards) database. As β-TCP and whitlockite have similar XRD profiles [26]–[28] diffractograms were compared to one obtained from a commercial 100% crystalline β-TCP (based on the manufacturer's description, ≥98% β-phase basis, Sigma-Aldrich Co., St Louis, MO, USA). The relative crystallinity (Cr) of the magnesium whitlockite powder was determined as described elsewhere [29]. In short, the most intense peak (31.4° at 2θ) of the powders was compared to the same peak of the reference β-TCP according to: 
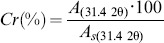
where Cr is the relative crystallinity of the measured magnesium whitlockite powder, and A_s(31.4θ)_ and A_(31.4θ)_ are the integrated area intensity of the 31.4 2θ peak of the β-TCP standard and the biomass powder respectively. TOPAS® software (Bruker AXS) was used for profile fitting and crystallite size calculations.

#### Fourier transform infrared spectroscopy (FTIR)

Attenuated Total Reflectance (ATR) Fourier transform infrared (FT-IR) spectra of pure powdered solids were obtained using a Bruker Tensor Series FT-IR (Bruker, Germany) spectrometer equipped with a zinc selenide crystal. Each Spectrum (sum of 64 scans) was collected from 4000 to 500 cm^−1^ at a spectral resolution of 4 cm^−1^. An air spectrum was also obtained at the beginning of the analysis to measure the water and carbon dioxide content in the air and these were subtracted from the sample spectra. The spectra obtained from both biotic and abiotic precipitates were compared with that of the commercial reference material β-tricalcium phosphate (β-TCP, ≥98% β-phase basis, Sigma-Aldrich Co., St Louis, MO, USA).

#### Scanning Electron Microscopy (SEM)

SEM imaging was carried out on two 30 days aged samples in order to compare: (1) the precipitate obtained from the sterile medium and (2) the precipitate that aged in the presence of *C. hydrogenoformans*. In both cases, a 40 mL sample was centrifuged, washed and dried, but the precipitate from the inoculated medium was also calcinated. Specimens were then mounted on SEM stubs with double side carbon tape. In order to avoid any interference during elemental analysis, no coating was applied. Examination and elemental analysis was done using a S-4700 Hitachi FE-SEM (Tokyo, Japan) working under vacuum at an acceleration voltage of 2.0 kV coupled to an Oxford INCA energy dispersive spectrometer (EDX) detector.

Backscattering electron (BSE) imaging was performed on an environmental SEM (ESEM, Quanta 200 FEG, FEI Company Hillsboro, OR) equipped with an energy dispersive X-ray (EDX) spectrometer (Genesis 2000, XMS System 60 with a Sapphire Si/Li Detector from EDAX Inc., Mahwah, NJ). Imaging was also done under the high vacuum mode of the ESEM microscope at an accelerating voltage of 20 kV and a working distance of 5–10 mm.

#### Transmission Electron Microscopy (TEM)

Whole mounts were prepared from 1 mL sample of an active 30 days bacterial culture of *C. hydrogenoformans* suspended in distilled water. They were imaged using a CM200 TEM (Philips, Netherlands), operating at an accelerating voltage of 200 kV. It was equipped with an AMT 2 k×2 k CCD Camera and an EDAX Genesis (EDAX Inc, Mahwah, NJ) energy dispersive spectrometer (EDS).

To document the evolution of the solids in the presence of the bacterial culture, a time course experiment was carried on a 27 days culture. Every 3 days, a 50 mL aliquot of medium was sampled and centrifuged. The resulting pellet was washed in a 0.1 M sodium cacodylate buffer and then fixed in l mL of fixative solution (2.5% glutaraldehyde in 0.1 M sodium cacodylate buffer). Samples were then centrifuged for 5 min at 5000 rpm and post-fixed with 1% aqueous OsO_4_+1.5% aqueous potassium ferrocyanide for 2 h, and washed 3 times with washing buffer. Samples were then dehydrated in a graded acetone series, infiltrated with graded Epon:acetone and embedded in Epon. Sections were polymerized for at least 120 h at 58°C. Sections that were 90–100 nm thick were cut using a diamond knife on a Reichert Ultracut II microtome, collected on 200-mesh copper grids, and stained with uranyl acetate and Reynold's lead for 6 and 5 min, respectively. Samples were imaged with a FEI Tecnai 12 transmission electron microscope (FEI Company, Hillsboro, OR) operating at an accelerating voltage of 120 kV equipped with an AMT XR-80C 8 megapixel CCD camera (Advanced Microscopy Techniques, Corp. Woburn, MA).

### Biomolecular techniques

To assess the potential role of proteins in the biomineralization process, protein extraction within and adsorbed to the precipitate was carried out on four independent cultures (200 mL each) after 21 days of *C. hydrogenoformans* growth. Each culture was centrifuged at 10000 rpm during 10 min at 4°C. The pellet was washed in 20 mL of sterile PBS buffer to remove any residual medium and then centrifuged. After its resuspension in a 10 mL crystal dissolving solution (151 U/mg trypsine in in 0.2 M EDTA), it was sonicated 5 times during 20 seconds at 40 Watts on ice using a Vibra-Cell Ultrasonic Processor (Sonics & Materials Inc., Danbury, CT, USA). This solution was then decanted for 1 hour and centrifuged. Potentially adsorbed proteins released in the supernatant were then analyzed by SDS-PAGE using a Criterion XT Precast Gel, 4–12% Bis-Tris (Bio-Rad, Hercules, CA, USA). SDS-PAGE was run at 200 V for 60 min in a Bio-Rad Criterion Cell. The running buffer was XT MOPS (Bio-Rad) and the gel was stained with the Bio-Rad Silver Stain Plus Kit according to the manufacturer's procedure. The same steps were also applied to samples drawn from the control experiments in the sterile DSMZ and inoculated modified DSMZ media.

## Results and Discussion

**Figure 1:**
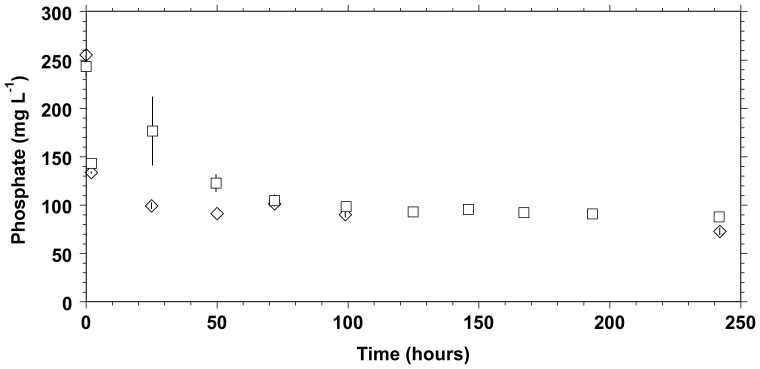
Change with time of dissolved total phosphate concentration in the sterile (dash) and inoculated (triangle) DSMZ medium after complementation with NaHCO_3_ (at time 0).

A white precipitate appeared immediately after addition of NaHCO_3_ to the DSMZ medium inoculated with *C. hydrogenoformans*. Its appearance coincided with a sharp decrease in the total phosphate concentration of the solution (Figure 1). The same phenomenon was noted following an addition of NaHCO_3_ to the same DSMZ medium without bacterial inoculation (hereafter referred to as the sterile DSMZ medium).

The centrifuged reaction product from 30-day culture in the DSMZ medium showed a 5.0 wt.% VSS/SS ratio, suggesting 95% inorganic precipitate and 5% biomass. This is inconsistent with the usual composition of microbial biomass where the inorganic portion represents typically less than 10% of dry weight [30]. Chemical analysis of the organic and inorganic components obtained from bacterial culture confirmed that 6.33% was organic (Table 1). This SS, expressed as absolute concentration, was 0.31+/−0.03 gSS.L^−1^ after 30 days, and was identical to what was measured within 2 hours following the initial addition of NaHCO_3_ in either sterile or inoculated media. The most abundant elements (in weight) were calcium and phosphorus (27 and 18% dry wt) while the total of other metals did not exceed 4% (Table 2). The metals detected were Mg, Mn, Cu, Ba and Al, with respective abundances of 3.81, 0.15, 0.13, 0.01, 0.01% dry wt. Converted to molar ratios, these relative abundances fall in the compositional range of the Ca-phosphate (CaP) phases listed in Table 2.

**Table 1:** Elemental analysis of a washed sample of *C. hydrogenoformans culture* grown on DSMZ medium compared to biomass elemental composition from literature [51].

Chemical element	Proportion (% wt)		Atomic fraction	
	This study	Literature	This study	Literature
C	2.81±0.09	48	1	1
H	0.85±0.03	7.3	3.62	1.8
N	0.49±0.02	11.3	0.15	0.2
O	2.18±0.14	32.5	0.58	0.5
S	0	0.01	0	0
Total	6.33±0.27	99.1		
Molecular weight (g⋅mol^−1^)			27	24.6

**Table 2:** Comparison of the elemental chemical composition of whitlockite [27], hydroxyapatite [52], octacalcium [53] to the elemental composition of suspended solids obtained after 39 days of *C. hydrogenoformans* growth on DSMZ medium. N.D.: not determined.

Chemical element	Proportion (% wt)			
	Suspended Solids	whitlockite	hydroxyapatite	octacalcium phosphate
Calcium (Ca)	27.30±1.70	33.91	39.9	32.63
Phosphorus (P)	17.95±1.16	20.38	18.5	18.91
Hydrogen (H)	N.D.	0.076	0.2	1.23
Oxygen (O)	N.D.	42.11	41.4	40.71
Metals	4.10±0.45	N.D.	N.D.	N.D.
Ca/P ratio (weight)	1.52±0.01	1.66	2.15	1.72
Ca/P ratio (molar)	1.17±0.01	1.28	1.66	1.33
Formula		Ca_9_(Mg, Fe^2+^)(PO_4_)_6_(PO_3_OH)	Ca_5_(PO_4_)_3_(OH)	Ca_8_H_2_(PO_4_)_6_.5H_2_O

The identical initial decrease, following medium complementation with NaHCO_3_, in phosphate concentration and the similarly steady pH maintained in sterile and inoculated media suggest that bacterial growth did not influence these parameters to induce the initial precipitation of the solid detected in our experiments. Within 25 hours of the initial formation of this precipitate, the dissolved phosphate concentration dropped slightly below 100 mg/L and varied very little for the remaining 29 days, in the sterile DSMZ medium. In contrast, measurements from several experiments on inoculated bottles showed a large scatter in values at 25 hours (resulting in the large error bar shown on Figure 1, at 25 hours) before a subsequent decline to a level comparable to the concentrations observed in the sterile medium after 48 hours.

Following repeated washing and recentrifugation, the precipitate in the sterile medium was invariably dissolved. This was not the case for the precipitate recovered from the inoculated DSMZ medium. The precipitate formed in the sterile DSMZ medium was therefore consistently more soluble in water.

**Figure 2:**
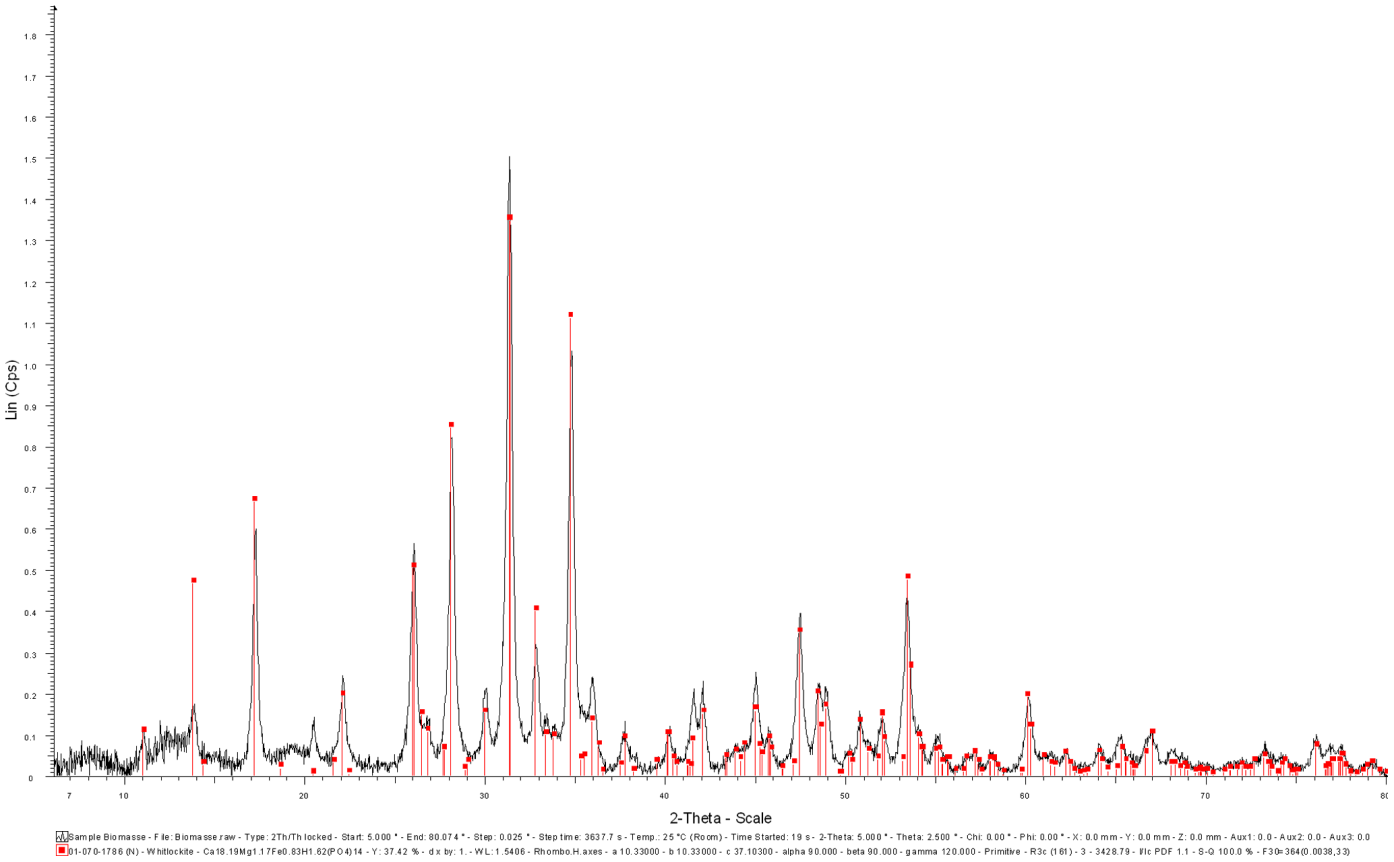
XRD spectra. Black: dried precipitate formed and obtained after 30 days of *C. hydrogenoformans* growth in the DSMZ medium. Red: whitlockite from the JCPDS (Joint Committee on Powder Diffraction Standards) database (number 01-070-1786).

**Figure 3:**
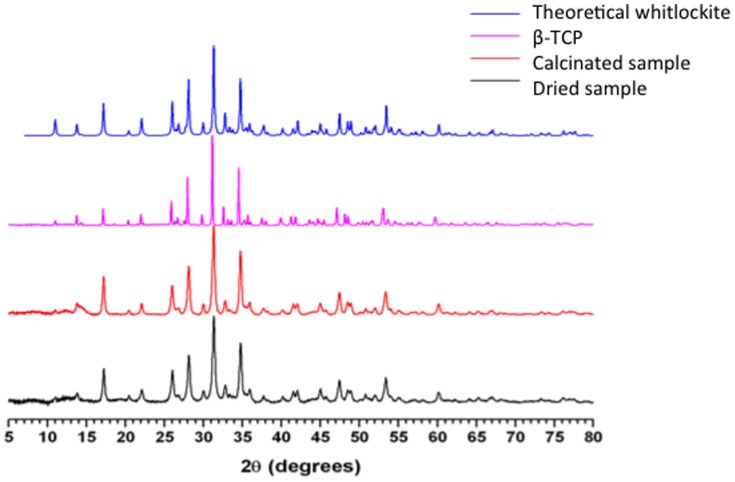
XRD spectra of the dried precipitate recovered after 30 days of *C. hydrogenoformans* growth in the DSMZ medium (identified as ‘Dried sample’), the dried sample after having been calcinated (identified as ‘Calcinated sample’), the commercial sintered β-TCP, and the whitlockite, calculated according to the JCPDS (Joint Committee on Powder Diffraction Standards) database (number 01-070-1786).

**Figure 4:**
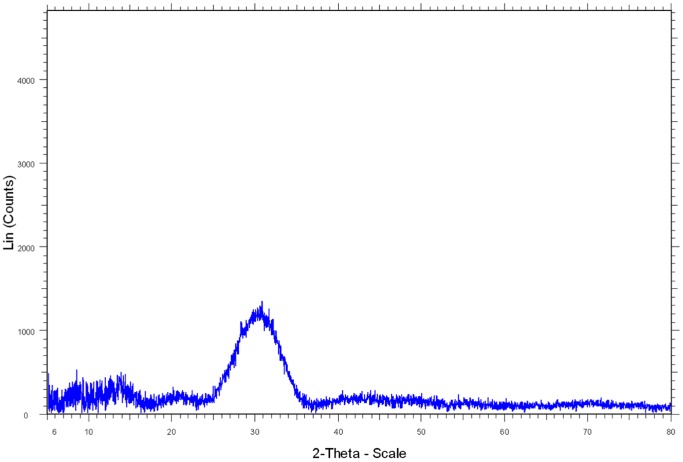
XRD pattern of the dried precipitate formed and sampled after 30 days of aging in the sterile DSMZ medium.

The XRD pattern (Figure 2) of solids recovered after 30 days in the inoculated DSMZ medium showed sharp peaks at the same angles as the one of whitlockite XRD pattern from the JCPDS database (file number 01-070-1786). Lattice parameters of these solids were determined as a = 10,330 A° and c = 37,103 A°, in agreement with those reported from natural whitlockite [26]. The calculated value of crystallite size was 30 nm compared to the crystallite size of 102 nm for the commercial β-TCP. The calculated crystallinity of the dried-only solid was 91.7%. After calcination (600°C) in air for 1 h, the solid showed 100% crystallinity (Figure 3). By comparison, the XRD spectrum (Figure 4) of the solid recovered after 30 days of aging in the sterile DSMZ medium showed a broad hump around 30° and no sharp peaks, suggesting either a total lack of crystallinity or barely incipient nanocrystallinity.

**Figure 5:**
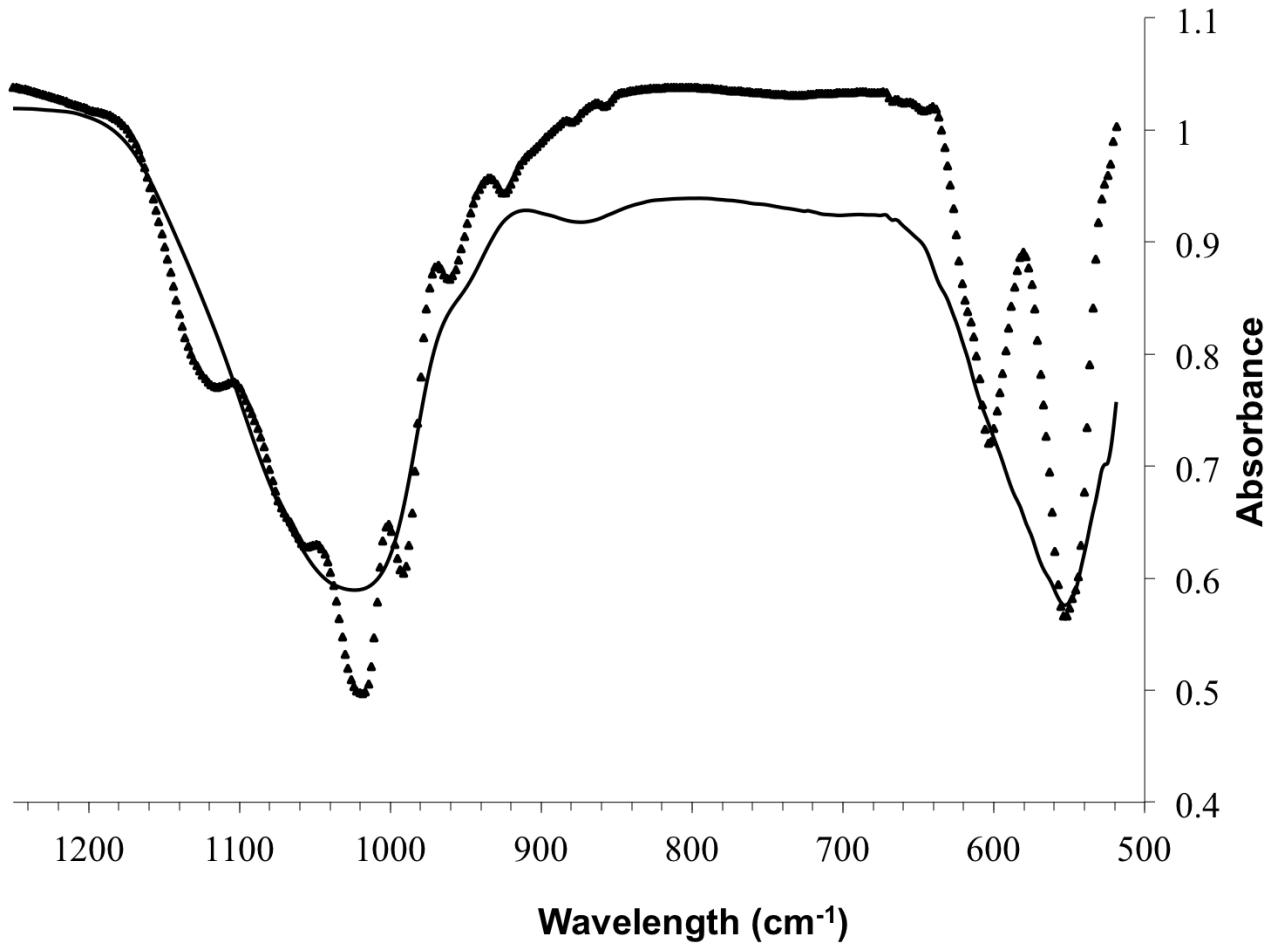
FTIR analysis of precipitate after 30 days of aging. Dried precipitate from sterile DSMZ medium (continuous line) and calcinated precipitate from the *C. hydrogenoformans* culture in the DSMZ medium (triangles).

The FTIR spectrum of the calcinated solids recovered from a 30 days experiment in inoculated DSMZ medium showed multiple split bands (Figure 5) associated with distinct absorption domains assigned to phosphate groups. Two groups of bands were observed: P–O stretching in HPO_4_ and PO_4_ groups at 1110, 1075, 1058, 1023, 962 603 cm^−1^ and the whitlockite specific bands at 990 and 555 cm^−1^. According to literature, these latter bands correspond to the phosphate groups with different structural environments present in whitlockite [31]–[33].

The FTIR spectra of the sample recovered from a sterile DSMZ medium (Figure 5) showed two broad and unsplit phosphate absorption bands between 1250 and 900 cm^−1^ and 650 and 500 cm^−1^. No bands related to carbonate groups were detected. Similar broad bands have been reported from FTIR spectra of amorphous calcium phosphate in previous studies where FTIR spectrum without any well-defined absorption bands, which indicated a disordered environment [34].

**Figure 6:**
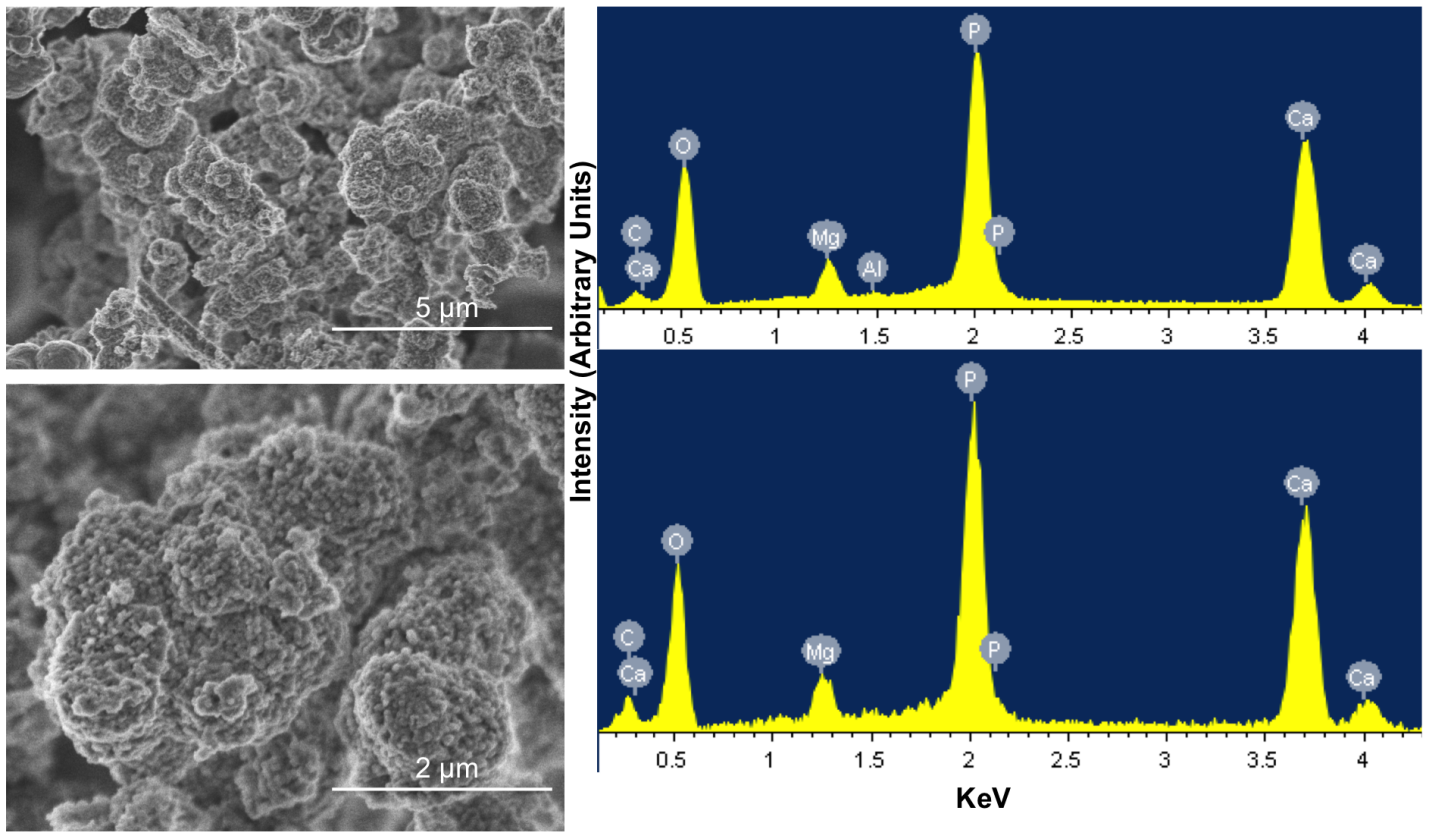
SEM-EDX analysis of two areas from a calcinated precipitate isolated after 30 days of *C. hydrogenoformans* growth in the DSMZ medium. Images on the left show two levels of magnification of same area. Images on the right show EDX spectrum of two distinct areas of the sample.

SEM imaging of solids from the 30-day inoculated DSMZ medium showed granules of 1–2 µm diameter consisting of angular particles approximately 50 nm across (Figure 6), which is a size consistent with the one determined from XRD spectra. Five EDS analyses from different granules revealed constant proportions of Ca (41), P (22), O (32), Mg (2) (% dry wt) without detectable spatial variation.

**Figure 7:**
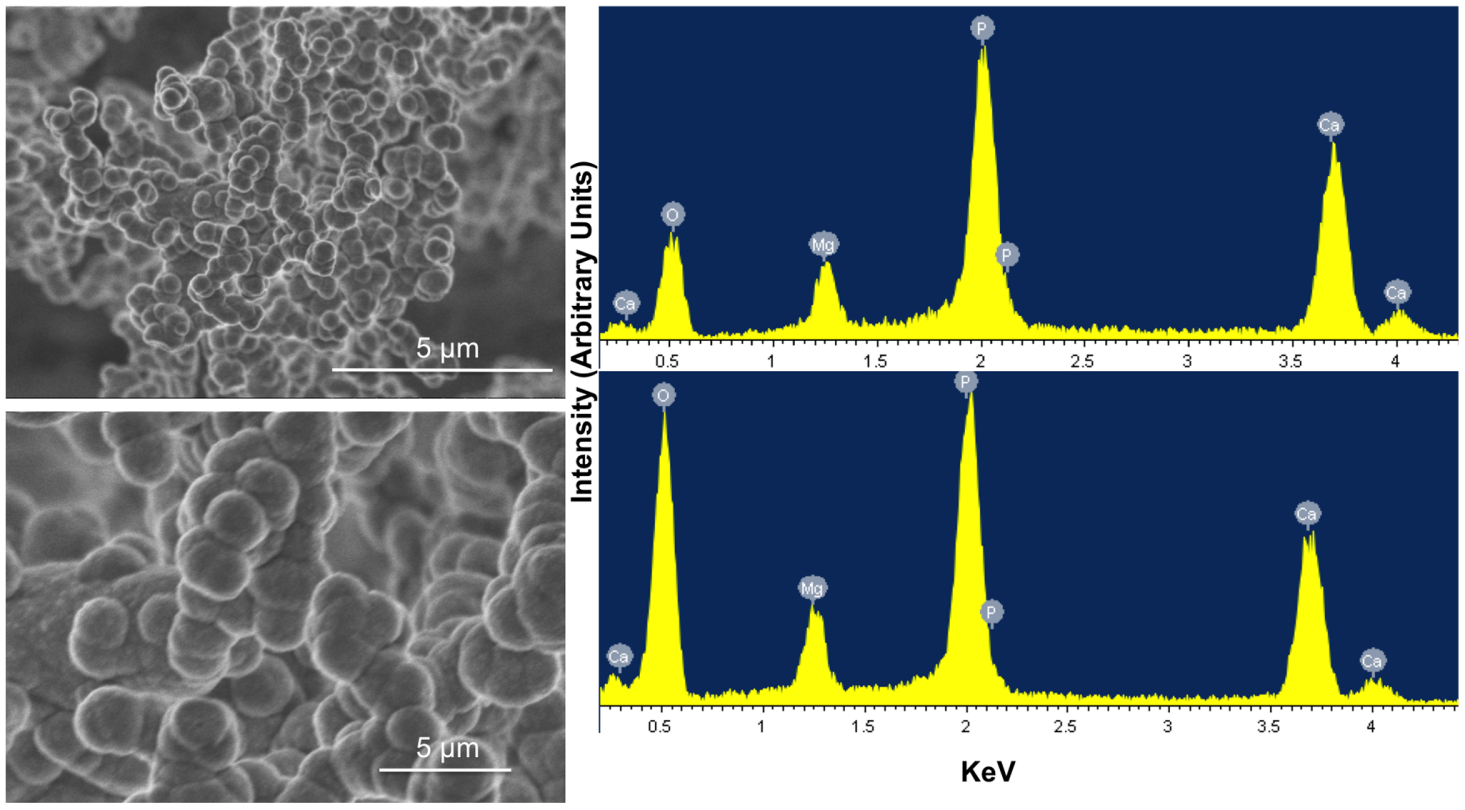
SEM-EDX analysis of two areas from a precipitate recovered and dried after 30 days of aging in the sterile DSMZ medium. Images on the left show two levels of magnification of same area. Images on the right show EDX spectrum of two distinct areas of the sample.

In contrast SEM imaging of the solid recovered from the sterile DSMZ medium revealed the presence of smooth spherical aggregates of 1–2 µm diameter (Figure 7). Their EDS analysis showed considerable variation in elemental composition within the following ranges: Ca (30–42), O (28–44) and Mg (4–5) while phosphorus remained constant around 24 dry wt.%.

**Figure 8:**
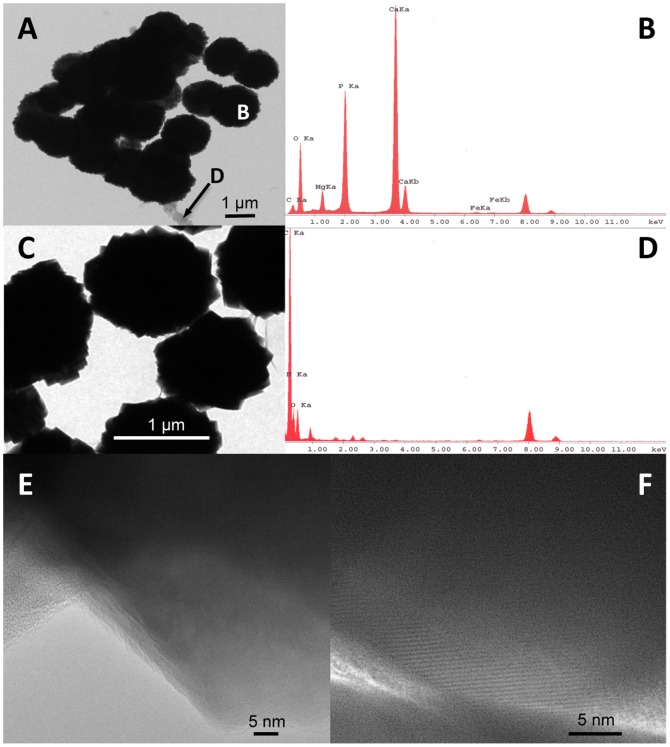
Images (A, C) and corresponding TEM-EDS analysis (B, D) of two areas in a whole-mount sample recovered after 30 days from the culture in the DSMZ medium. (C) Magnification of the area in (A) showing biofilm covering and binding the granules, (B) Spectrum from the granule labelled B. (D) Spectrum from the organic material labelled D. (E, F) HR-TEM images of the granules' edges showing lattice fringes.

To document the evolution of CaP phases with time, from an amorphous precursor phase to crystalline phases, samples incubated in the presence of *C. hydrogenoformans* were analysed using analytical TEM and biochemical techniques. An unstained whole-mount (see TEM analysis in methods) of a sample from the 30-days inoculated DSMZ medium showed granules made of angular particles similar in size and shape to those imaged by SEM. EDX confirmed their uniform concentrations of P, Ca and Mg (Figure 8, A, B). The granules were covered with a biofilm (Figure 8, C). Lattice fringes (Figure 8, E, F) were observed at the edges of the granules, confirming the crystalline character of their constituent particles.

**Figure 9:**
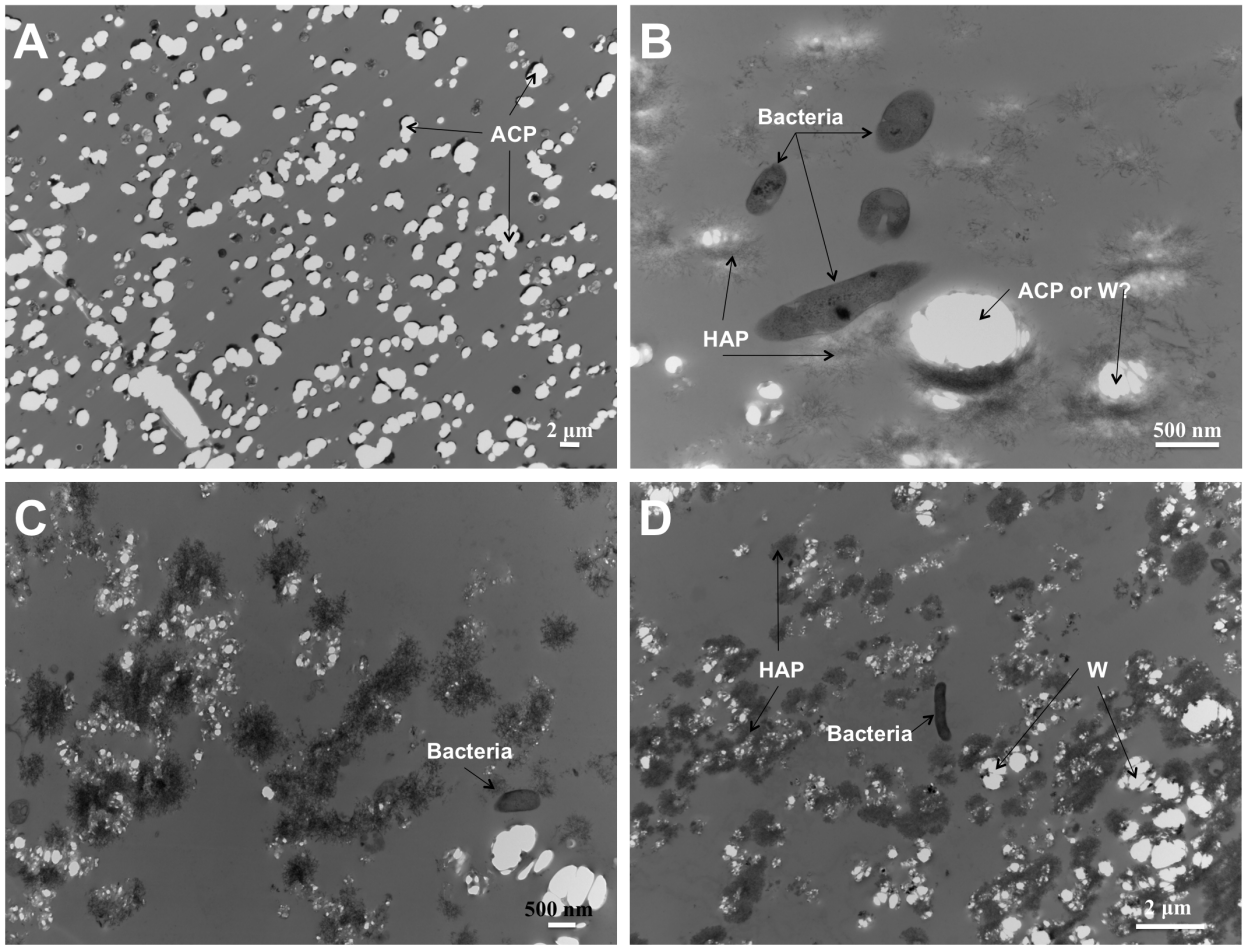
TEM imaging of solids identified in sterile and inoculated DSMZ media. (A) Sample recovered from the sterile DSMZ medium after 15 days of aging. ACP granules embedded in resin are visible. (B, C, D) Images from samples recovered after 3, 8 and 15 days respectively from a time course experiment in inoculated DSMZ medium. Three solid phases are distinct and interpreted to be either amorphous CaP (ACP) or whitlockite (W) and nanocrystalline hydroxyl-apatite (HAP). Bacteria (B) are also visible.

**Figure 10:**
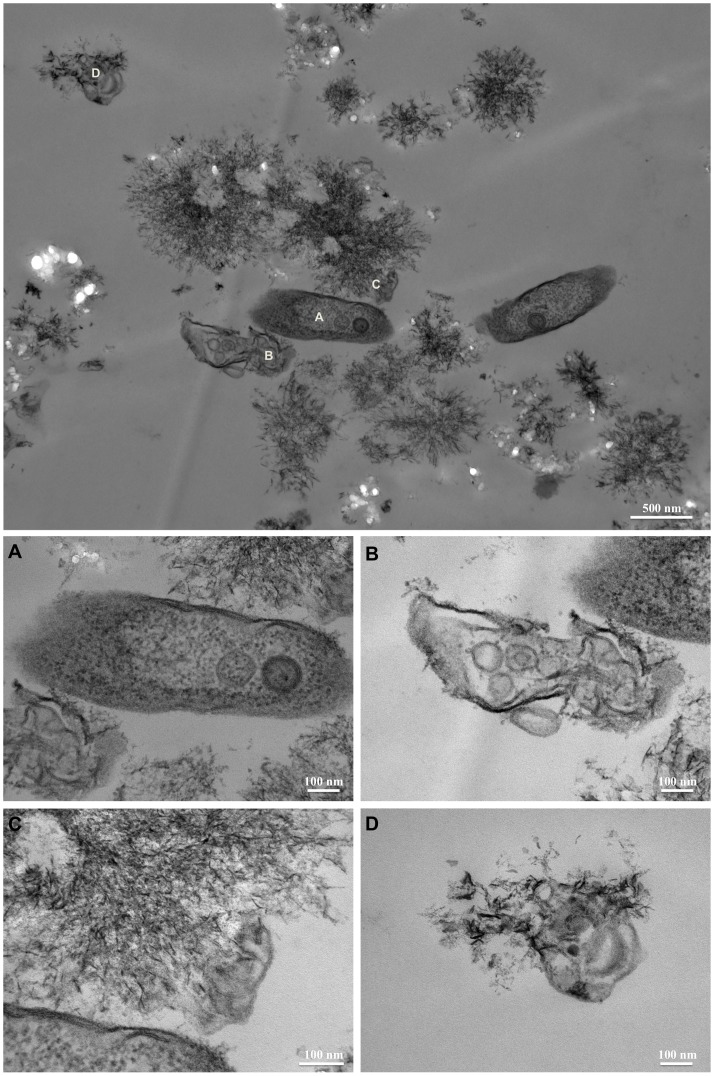
TEM imaging of the inoculated DSMZ medium sampled after 6 days of culture. A to D show magnification of cell lysis and spatial association of the lysed vesicle of *C. hydrogenoformans* and the interpreted HAP.

**Figure 11:**
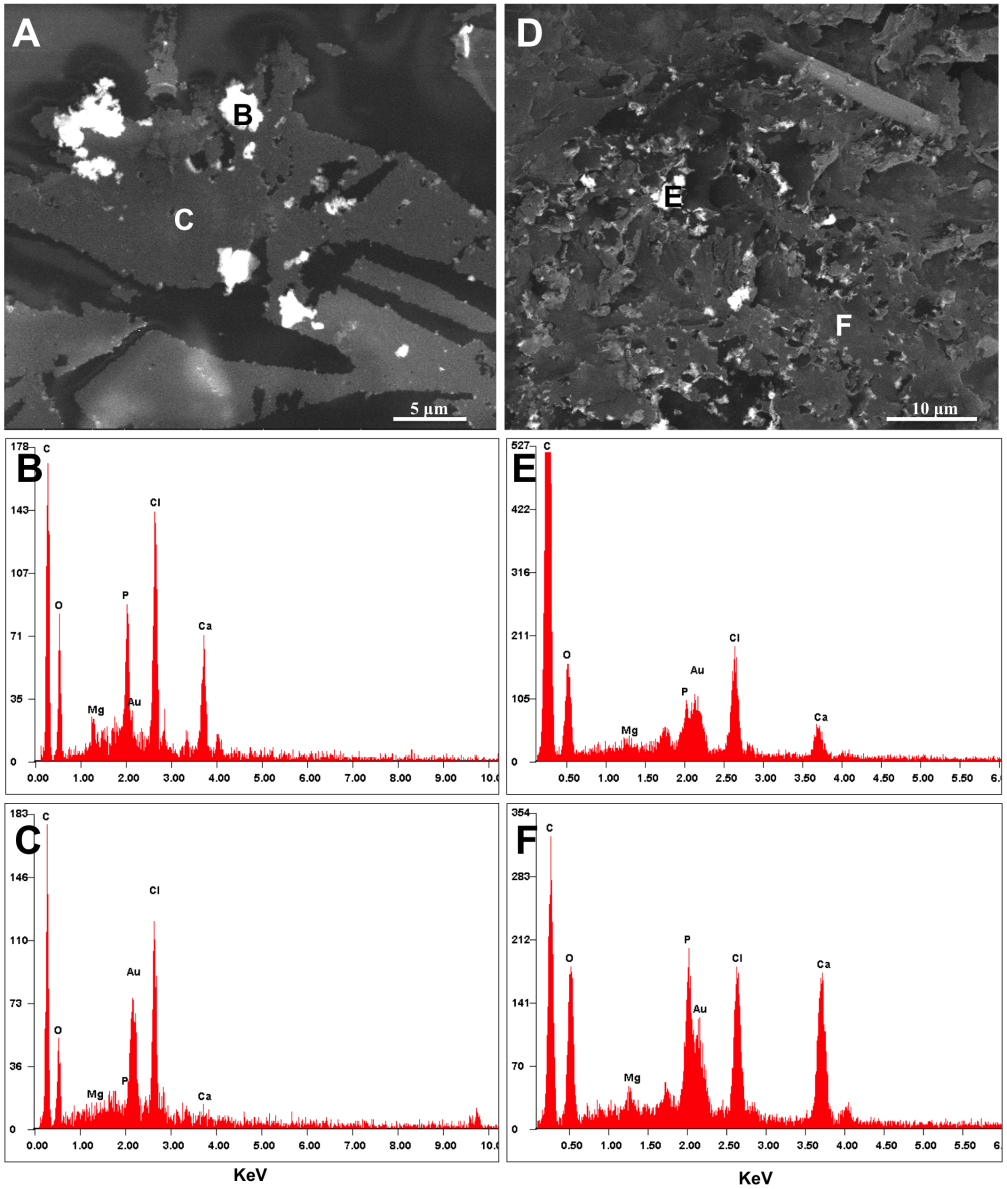
Backscatter electron image and EDS analysis by SEM of samples recovered after 15 days, also shown in Figure 9. (A) Sample recovered from sterile DSMZ medium, and (B) EDS analysis of its precipitate. (C) EDS analysis of the embedding epoxy matrix. (D) Sample recovered after 15 days of *C. hydrogenoformans* growth in the DSMZ medium, and (E) EDS analysis of its solid precipitate, (F) EDS analysis of its embedding epoxy matrix showing that Ca and P are present.

Ultrathin sections of samples obtained from the time course experiment shed additional light on the evolution of the amorphous precipitate. Because of its high solubility, the only evidence of the solid granules produced in the sterile DSMZ medium were holes left in the epoxy matrix (Figure 9, A). The granules were dissolved during the sectioning process, which exposed them to low-pH water. After 3 days of *C. hydrogenoformans* growth, a nanocrystalline phase composed of 30–50 nm rod-like crystals, distinct from the previously characterized whitlockite, was observed (Figure 10, D). These nanorods resembled hydroxyapatite produced by bacteria and mammalian cells such as bone and calcified tissue [35]. In this sample, well-preserved bacteria were observed (Figure 9, B). Backscatter analysis was carried out on the cutting face of the epoxy blocks used for the sectioning. Distribution and chemical composition of the amorphous CaP precursor confirmed the results previously obtained with TEM and SEM analysis (Figure 11, A, B). No traces of CaP material were detected in the epoxy matrix for the sample recovered after 15 days from the sterile DSMZ medium (Figure 11, C). The backscatter analysis of the blocks containing the sample recovered after 15 days of incubation in an inoculated DSMZ medium (Figure 11, D) revealed a presence both larger crystals and the chemical signature of a CaP material dispersed throughout the matrix, which could be mixture of disaggregated granular whitlockite and nanorods (Figure 11, E, F). With time, there was increasing visible evidence for bacterial lysis (Figure 10) and the nanorods were always associated with those degraded bacterial remnants (Figure 10, C, D). Disruption of cytoplasmic membrane led to the formation of vesicles that could have served as nucleation site for the precipitation of hydroxyapatite (Figure 10, A, B).

In summary, we report the formation of two distinct CaP crystalline solids in the presence of *C. hydrogenoformans* grown in a phosphate and calcium rich medium under a near-steady pH and at a controlled temperature. For both CaP phases, the path of biomineralization appears to follow biologically induced mineralization (BIM) [7] in contrast to biologically controlled mineralization (BCM) [8]. Both phases only appeared in the inoculated DSMZ medium.

One mode of mineralization involves conversion of a granular amorphous CaP precipitate to polycrystalline granules of whitlockite. The presence in the whole mount of a microbial biofilm that covers and binds the granules suggests that the biofilm creates the conditions for a dissolution-reprecipitation mechanism. This is strongly supported by the phosphate resolubilization (Figure 1) observed during the first 25 hours following DSMZ medium inoculation with *C. hydrogenoformans*. It has been reported elsewhere that whitlockite formation was caused by the binding of the amorphous CaP precursor with phospholipids, with the magnesium content in the precursor inhibiting apatite to the benefit of whitlockite formation [36]. In our system, the bacterial biofilm could have played a similar role in the conversion of amorphous CaP to whitlockite.

A number of biochemical analysis were conducted on the precipitate (see Biomolecular techniques in methods section) to look for evidence of biomolecules occluded or strongly adsorbed to its surface. The analyses of metabolic by-products of bacteria (mono or disaccharides, VFAs and alcohols) and protein content in the crystalline material dissolved to detect them were negative. Since this precipitate, according to XRD, is dominantly whitlockite (or a Mg-stabilized β-TCP), we conclude to a conversion by a biologically induced mechanism rather than a biologically controlled one. The inhibiting effect of Mg on a conversion from an amorphous CaP precursor to hydroxyapatite is widely documented [37], [38]. It is therefore plausible that the biofilm counteracts this by chelating Mg and decreasing its capacity to inhibit either dissolution of the amorphous CaP or nucleation of other crystalline CaP phases.

The second mode of mineralization involves formation of nanorods interpreted to be hydroxyapatite-like (HA). The bacterial cells were lysed and fragmented leading to formation of vesicles, and there was a direct association between the HA nanorods and the fragmented membrane material (Figure 9, B, C, D; Figure 10; Figure 11, F). According to Mann [6], a cellular membrane can serves as a template for the nucleation of HA. Our nanorods resemble closely those obtained in other cases of induced biomineralization described in *Serratia* species, where presence of high concentration of calcium and phosphate in the growth medium coupled to the presence of an organic matrix (EPS), triggered hydroxyapatite nucleation [39].

Bioresorption or biodegradation of the CaP ceramics is a biological mechanism during which part of (or all) grafted CaP disappear partially (or completely) over a period of time *in vivo* [40]. The major factor accelerating CaP resorption is local pH diminution, which can be caused either chemically or biologically [41]. This feature allows avoiding lifetime implants of foreign bodies and stronger newly formed bone [40]. All synthetic calcium phosphate ceramics are bioresorbable to a certain extent, from most to least: amorphous calcium phosphate, tetracalcium phosphate, α-TCP, β-TCP, hydroxyapatite (HA) [17]. Resorption rate increases with surface area increase and decreases with an increase of crystallinity, grain size and ionic substitutions of CO_3_^2−^, Mg^2+^ and Sr^2+^ in HA [41]. Little is known about bioresorption of whitlockite. A previous study has concluded that, in vivo, whitlockite was biodegraded at a faster rate than HA - because of differences in density and pore diameter - but at a slower rate than β-TCP, due probably to the presence of metal oxides that may make the material less resorbable [42]. Magnesium incorporation was also shown to stimulate human osteoblast proliferation [43] making whitlockite less quickly resorbable than β-TCP but more osteoinductive, thus with an interesting range of application in bone engineering.

## Conclusion

*C. hydrogenoformans* is a carboxydotrophic bacterium that was first isolated from a hot spring in Kunashir Island, Russia [44]. This bacterium has been subject to an extensive investigation with respect to its genomic and metabolic activities [44]–[47]. The main interest in this organism has been its ability to produce hydrogen from carbon monoxide, which is a potential biological based alternative to the currently conventional chemical catalysed water-gas shift reaction [48]–[50]. So far, there has not been any report on biomineralization activities associated with *C. hydrogenoformans*. The results presented here show two previously unknown modes of biomineralization carried out in the presence of *C. hydrogenoformans*.

The suggested BIM pathway for HAP nanorods follows a typical pattern, widely documented in the literature [7], but the association of nanorods with cellular components resulting from *C. hydrogenoformans* lysis is unusual. The whitlockite, however, is a novel aspect where a biofilm is involved but acts on an amorphous precipitate, by a different mechanism possibly neutralizing the inhibiting effect of Mg on the dissolution of the amorphous CaP and the nucleation of whitlockite. The result is a biphasic crystalline product induced by the bacterial activity and decay. Further investigation of the mechanisms by which this biomineralization proceeds could lead to interesting applications in the field of CaP bioceramics.
